# Supplementary material for: Modeling glioblastoma heterogeneity as a dynamic network of cell states
Source: Mol Syst Biol. 2021 Sep 16;17(9):e10105. doi: 10.15252/msb.202010105 (PMC8444284; doi:10.15252/msb.202010105)
Supplement: Supplementary file 5 — Source Data for Figure 3 [file MSB-17-e10105-s001.zip › Figure3A_sourcedata/GSEA_3065/hallmarks_state4.GseaPreranked.1623416296068/HALLMARK_ANGIOGENESIS.html]

Details for gene set HALLMARK\_ANGIOGENESIS[GSEA]

|  || Dataset | state4 |
| Phenotype | NoPhenotypeAvailable |
| Upregulated in class | na\_neg |
| GeneSet | HALLMARK\_ANGIOGENESIS |
| Enrichment Score (ES) | -0.65239716 |
| Normalized Enrichment Score (NES) | -1.7314835 |
| Nominal p-value | 0.00792393 |
| FDR q-value | 0.006523197 |
| FWER p-Value | 0.062 |
Table: GSEA Results Summary

  

Fig 1: Enrichment plot: HALLMARK\_ANGIOGENESIS      
 Profile of the Running ES Score & Positions of GeneSet Members on the Rank Ordered List

  

| PROBE | GENE SYMBOL | GENE\_TITLE | RANK IN GENE LIST | RANK METRIC SCORE | RUNNING ES | CORE ENRICHMENT || 1 | TNFRSF21 |  |  | 97 | 0.251 | 0.0706 | No |
| 2 | FSTL1 |  |  | 1367 | 0.068 | -0.1094 | No |
| 3 | STC1 |  |  | 2141 | 0.043 | -0.2185 | No |
| 4 | MSX1 |  |  | 2143 | 0.043 | -0.2040 | No |
| 5 | CCND2 |  |  | 2367 | 0.038 | -0.2267 | No |
| 6 | PDGFA |  |  | 2529 | 0.034 | -0.2408 | No |
| 7 | COL5A2 |  |  | 2672 | 0.031 | -0.2528 | No |
| 8 | LPL |  |  | 3121 | 0.023 | -0.3167 | No |
| 9 | VEGFA |  |  | 3787 | 0.001 | -0.4228 | No |
| 10 | VCAN |  |  | 4158 | -0.019 | -0.4755 | No |
| 11 | JAG1 |  |  | 4496 | -0.044 | -0.5143 | No |
| 12 | FGFR1 |  |  | 4568 | -0.049 | -0.5089 | No |
| 13 | NRP1 |  |  | 4994 | -0.082 | -0.5487 | No |
| 14 | APP |  |  | 5192 | -0.101 | -0.5457 | No |
| 15 | ITGAV |  |  | 5309 | -0.110 | -0.5265 | No |
| 16 | LRPAP1 |  |  | 6096 | -0.294 | -0.5514 | Yes |
| 17 | TIMP1 |  |  | 6191 | -0.409 | -0.4259 | Yes |
| 18 | SPP1 |  |  | 6231 | -0.520 | -0.2536 | Yes |
| 19 | POSTN |  |  | 6256 | -0.753 | 0.0011 | Yes |
Table: GSEA details [plain text format]

  

Fig 2: HALLMARK\_ANGIOGENESIS: Random ES distribution      
 Gene set null distribution of ES for **HALLMARK\_ANGIOGENESIS**
